# Evaluation of a Sensor System for Detecting Humans Trapped under Rubble: A Pilot Study

**DOI:** 10.3390/s18030852

**Published:** 2018-03-13

**Authors:** Di Zhang, Salvatore Sessa, Ritaro Kasai, Sarah Cosentino, Cimarelli Giacomo, Yasuaki Mochida, Hiroya Yamada, Michele Guarnieri, Atsuo Takanishi

**Affiliations:** 1 Graduate School of Advanced Science and Engineering, Waseda University, Tokyo 169-8555, Japan; gwlrzd@fuji.waseda.jp (D.Z.); ritaro.kasai@gmail.com (R.K.); sarah.cosentino@aoni.waseda.jp (S.C.); 2 Hibot Corporation, Watanabe Corporation Building 4F, 5-9-15 Kitashinagawa, Shinagawa-ku, Tokyo 141-0001, Japan; sessa@hibot.co.jp (S.S.); cimarelli@hibot.co.jp (C.G.); mochida@hibot.co.jp (Y.M.); yamada@hibot.co.jp (H.Y.); guarnieri@hibot.co.jp (M.G.); 3 Department of Modern Mechanical Engineering, Waseda University, Tokyo 169-8555, Japan; 4 Humanoid Robotics Institute (HRI), Waseda University, Tokyo 162-0044, Japan

**Keywords:** life detection, earthquake rescue, gas sensor, voice recognition, thermal vision camera

## Abstract

Rapid localization of injured survivors by rescue teams to prevent death is a major issue. In this paper, a sensor system for human rescue including three different types of sensors, a CO_2_ sensor, a thermal camera, and a microphone, is proposed. The performance of this system in detecting living victims under the rubble has been tested in a high-fidelity simulated disaster area. Results show that the CO_2_ sensor is useful to effectively reduce the possible concerned area, while the thermal camera can confirm the correct position of the victim. Moreover, it is believed that the use of microphones in connection with other sensors would be of great benefit for the detection of casualties. In this work, an algorithm to recognize voices or suspected human noise under rubble has also been developed and tested.

## 1. Introduction

During the 21st century, more than 522 significant earthquakes happened [1], with a death toll of more than 430,000 worldwide [2]. The majority of deaths are caused by buildings collapsing and trapping occupants under the rubble. In fact, if the casualty is an uninjured, healthy adult with a supply of fresh air, then they can survive for about 72 h. Eighty percent of survivors can be rescued alive within 48 h of a collapse, but after 72 h the survival rate reduces exponentially [3]. This time limit can be much shorter due to air supply shortage, environmental temperature, the health condition of the casualty, etc. Therefore, to reduce mortality after a natural disaster, the rapid detection of survivors inside collapsed structures is of the utmost importance. The current searching method is based on survivors’ testimony to establish the possible presence of casualties under the rubble. Rescue operations are generally carried out in subsequent steps. First, the rescue team accesses the area with dogs to search for casualties on the surface. Then, the rescue team uses video cameras to check the situation under the rubble. Finally, the rescue team tries to verify the presence of people trapped under the rubble [4]. However, the first objective of the rescue team is to assess two essential characteristics of the searching area: the existence of a sufficient number of survival spaces, and the stability of volume of the ruins [5]. This assessment is subjective and prone to change due to structural instability and the unknown situation under the rubble. Accessing collapsed structures is extremely dangerous for rescue teams because subsequent aftershocks might furthermore undermine the stability of structures. Moreover, rescue workers are at great risk for the development of physical, cognitive, emotional, or behavioral symptoms of stress [6]. Hence, rapid localization of survivors under the rubble, avoiding direct access and exploration of the affected area, is essential for rescue teams.

To reduce the risks of rescue operations and accelerate the localization of casualties, several methods based on the use of sensor technologies have been proposed. Currently, rescue teams use life detection systems mainly based on microphones, optical/thermal cameras, and Doppler radar [7]. Audio signal analysis is an effective method to detect humans trapped under rubble, and some systems are already commercially available, such as the Acoustic Life Detector, which is based on audio signal processing to identify victims’ low-frequency sounds. Moreover, several refined audio processing algorithms have been developed to detect human presence [8,9,10]. However, microphones become less accurate in the case of high background noise such as pneumatic drills, breakers, vehicles, wind, power cables, and water flows that can be present in a real scenario. Another limitation of audio detection systems is that they cannot locate unconscious victims.

Cameras are also widely used in rescue operations. Cameras are often mounted on mobile robots to explore dangerous and inaccessible areas because they are an efficient interface for human rescue [11,12,13,14]. Some researchers proposed thermal cameras to detect trapped humans to overcome the problems of limited visibility under the rubble [15,16]. However, even though cameras are an efficient method to detect casualties, their effectiveness is limited by their inherent reduced angle of view, the presence of obstacles, and the generally limited visibility under the rubble. In a real scenario, rapid localization and accurate estimation of the person’s position are fundamental for an efficient rescue operation, and images alone do not provide enough information. 

Doppler radar has been widely used in disaster rescue operations due to its efficiency in detecting motion behind obstacles [17]. In fact, frequency or phase shift in a reflected radar signal can be used to detect motions of only a few millimeters such as heartbeat or breathing [18]. However, Doppler radar requires accurate calibration and even small environmental changes due to aftershocks and structural instability have a negative impact on the performance of this kind of system [19]. Moreover, due to its narrow-angle view, this system is not suitable for wide disaster areas.

The use of gas sensors for human detection via analysis of changes in carbon dioxide (CO_2_) and oxygen (O_2_) in the environment due to human breath has also been proved feasible [3]. However, this system and several other experimental sensor systems for life detection have only been tested in controlled laboratory settings [20,21,22,23,24,25].

The objective of this study is to evaluate the performance of a system based on three different sensors in detecting live human presence under the rubble in a high-fidelity simulated disaster area in the open.

The system was composed of these three types of sensors:

1. Gas sensors (O_2_ and CO_2_) for the detection of human breath and quality of air.
2. Microphones for the detection of voices, human-produced sounds, or environmental noise.
3. Thermal vision camera for a direct view of the environment, localized temperature patterns.

The only a priori information during the experiment was that one person, and only one, was present in the area.

The article follows this structure: Section 2 introduces the sensors being tested, the specific sound recognition algorithm used, the data analysis method, and the experimental protocol. Section 3 and Section 4 present the results and performance evaluation for each sensor. The last section summarizes the results and proposes future work.

## 2. Materials and Methods

In this section, we describe the sensors being tested, the sound recognition algorithm, the data analysis method, and the experimental protocol.

### 2.1. Gas Sensors (CO_2_ and O_2_ Sensors)

The FIGARO TGS4161 CO_2_ sensor was chosen for its high sensitivity. This sensor can detect CO_2_ in a range of 350~10,000 ppm. Moreover, this sensor exhibits a linear relationship between the change in electromotive force and CO_2_ gas concentration on a logarithmic scale and shows excellent durability against the effects of high humidity.

The FIGARO SK-25F O_2_ sensor was chosen. The advantage of this sensor is that it is not influenced by other gases such as CO_2_, CO, and H_2_S that can be present in the environment. It shows a good linearity up to 30% O_2_, inside the measurement range in the real disaster area, and has chemical durability. 

These two sensors were connected to a Waspmote motherboard from Libelium Comunicaciones Distribuidas S.L. (Zaragoza, Spain). The motherboard transmits the data stream via USB to a PC for data storage and analysis every 10 s. The CO_2_ sensor needs 10 min of warm-up time to stabilize its data output. The O_2_ sensor does not need an initial warm-up. The Waspmote board was mounted on a long telescopic pole and the pole was introduced in the gaps in the rubble for more than two minutes, then the collected CO_2_ and O_2_ data were analyzed.

### 2.2. Thermal Vision Camera

**Figure 1:**
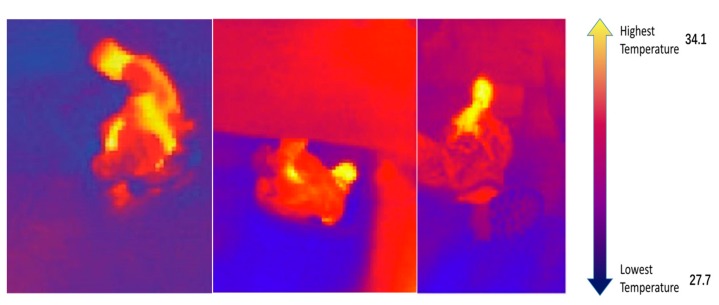
Three images from thermal camera from different directions.

The LEPTON thermal camera from FLIR (Wilsonville, OR, USA), which is a complete long-wave infrared (LWIR) camera, was chosen. Its size is 8.5 × 11.7 × 5.6 mm (without socket). The lens horizontal range is 56 degrees, the diagonal range is 71 degrees, and the resolution is 160 × 120 active pixels. The images are sent in streaming to PC via LAN communication using a Hi-Bot Corp. TITech M4 Controller as grabber. Dedicated software visualized the image data automatically, adapting the temperature range to a red–blue color map. The software also estimated the highest and lowest temperature in the image (Figure 1).

The thermal camera was mounted on another telescopic pole; the pole was introduced in the gaps in the rubble and manually rotated to check the surrounding environment under the rubble. In Figure 1, a thermal image of an object with an outline similar to a human is shown. When a human-like thermal outline is detected, the affected area is tested from different angles and directions to verify if it really is a human victim.

### 2.3. Microphone

#### 2.3.1. *Hardware and Audio Signal Process*

The low-energy Bluetooth SONY ECM-AW4 microphone was chosen. This is a non-directional microphone with a frequency response in the range of 300–9000 Hz.

To discriminate human voice from environmental noise, six voice features, usually used for voice detection, have been computed with MatLab.

● Energy Entropy 

Entropy is a measure of state unpredictability. The definition of entropy *H* of a discrete random variable *X* with possible values *x_i_* and probability mass function *P(X)* is:

(1) $$H\left(X\right)=-\sum_{i=1}^{n}p\left(x_{i}\right)\log p\left(x_{i}\right).$$

● Signal Energy

The energy *E_s_* of a continuous-time signal *x(t)* is defined as:

(2) $$E_{s}=\int_{-\infty }^{\infty }{\left|x\left(t\right)\right|}^{2}dt.$$

● Zero Crossing Rate

The rate of sign changes of a signal, a useful parameter of Voice Activity Detection (VAD) [26]:

(3) $$ZCR=\;\frac{1}{T-1}\sum_{t=1}^{T-1}1_{R<0}\left(s_{t}s_{t-1}\right),$$

where s is a voice single of length *T* and 1*_R<0_* is an indicator function.

● Spectral Roll-Off

The roll-off frequency is defined as the frequency under which a percentage (85% cutoff) of the total energy of the signal spectrum is contained.

(4) $$\sum_{n=1}^{R_{t}}M_{t}\left[n\right]=0.85\times \sum_{n=1}^{N}M_{t}\left[n\right],$$

where $M_{t}\left[n\right]$ is the magnitude of the Fourier transform at frame t and frequency bin *n*, and $R_{t}$ is the frequency.

● Spectral Centroid

The Spectral Centroid *C* is calculated as the weighted mean of the frequencies present in the signal, determined using an FFT with their magnitudes as the weights [27]. If *x(n)* represents the weighted frequency value, or magnitude, of bin number *n*, and *f(n)* represents the center frequency of that bin, the Spectral Centroid *C* is:

(5) $$C=\frac{\sum_{n=0}^{N-1}f\left(n\right)x\left(n\right)}{\sum_{n=0}^{N-1}x\left(n\right)}.$$

● Spectral Flux

Spectral Flux is a measure of how fast the power spectrum of the signal is changing, comparing the power spectrum of one frame with the power spectrum of the previous frame:

(6) $$F_{t}=\sum_{n=1}^{N}{\left(N_{t}\left[n\right]-N_{t-1}\left[n\right]\right)}^{2},$$

where $N_{t}\left[n\right]\;$and $N_{t-1}\left[n\right]$ are the normalized magnitude of Fourier transform at frames *t* and *t*−1.

The audio signal was divided into non-overlapping frames of 10 ms and for each frame the above six features and their statistical deviation are calculated. In particular, for Energy Entropy, Zero Crossing Rate, Spectral Roll-off, and Spectral Centroid, the Standard Deviation has been computed, while for Signal Energy and Spectral Flux, the Standard Deviation by Mean Ratio has been computed. These six statistical values are the final feature values that characterize the audio signal.

#### 2.3.2. *Human Voice Detection Algorithm*

The human voice detection algorithm was based on Support Vector Machine (SVM) from MatLab and consisted of a training phase and a classification phase. The Hard-margin SVM [28] classifies data identifying the best hyperplane that divides all data points into two groups [29,30].

● Training Phase

A database composed of 1588 samples of speech voice files, including male and female voices speaking in several languages, and 1687 samples of environment noise files including different types of environmental noise was created. All the sound samples were pre-processed with a bandpass filter (50 Hz~3000 Hz). The six statistical audio features were computed for each sound sample, and arranged in two matrices, a 6 × 1588 matrix for human voice samples and a 6 × 1687 matrix for environmental noise samples. These matrices were used in the SVM based algorithm as training data. 

● Classification Phase

**Figure 2:**
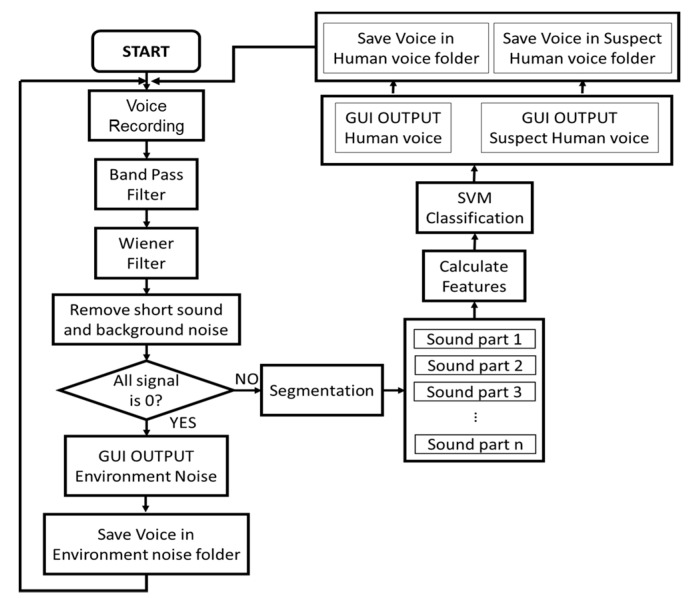
SVM classification for human voice and environment noise.

The flow chart of the classification phase is shown in Figure 2. Its fundamental steps are:

1. Voice recording phase: the system records voice at 5-s intervals.
2. Recorded data are bandpass filtered (50 Hz~3000 Hz)
3. Data are filtered with a Wiener filter. The Wiener filter minimizes the Mean Square Error (MSE) between the estimated random process and the desired operation. This filter is generally used to remove noise from a recorded voice.
4. Short sounds and background noise are removed. First, an adaptive threshold to remove background noise has been used. The reference level of environmental noise must be calculated. As the noise in the disaster area is high and highly variable, an adaptive background noise reference has been defined according to the equation:
  (7) $$Ref_{noise}\;=\alpha Vol_{t}+\left(1-\alpha \right)Vol_{t-1}$$
  where α is the smoothing factor of $Ref_{noise}$ change, $Vol_{t}$ is the average volume [dB] of current 5 s voice data, $Vol_{t-1}$ is the volume of previous 5 s voice data. It has been empirically found that a = 30% yields the best performance. Then, if the volume of the sound sample is lower than 1.3 times $Ref_{noise}$, the algorithm identifies the sound sample as environmental noise and discards it. Sound signals that are 1.3 times higher than $Ref_{noise}$ are suspect sounds. Then, the algorithm checks the length of this suspect sound. As human voice sound is assumed to last more than 300 ms, sounds shorter than 300 ms are removed. After removing short sounds, this suspect sound is processed with SVM to identify possible human noise.
5. Segmentation. The 5-s audio signal, after removing short sounds and background noise, is broken into shorter audio samples of 10 ms.
6. Audio statistical features, as described in Section 2.3.1, are computed for these shorter 10-ms audio samples.
7. SVM Classification. Sounds are differentiated in human voice or noise.

### 2.4. Experiments

#### 2.4.1. Experimental Environment

**Figure 3:**
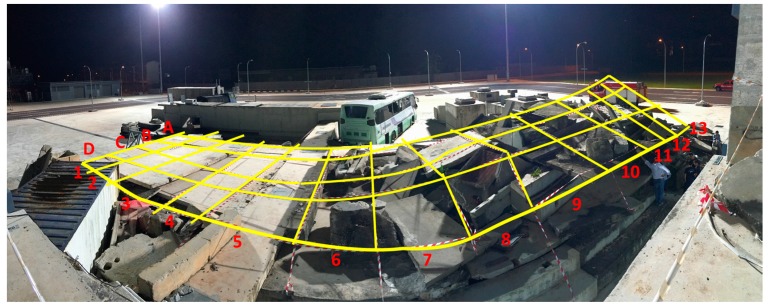
Experiment environment (panorama image).

The tests were conducted at a site at the Singapore Civil Defence Force (SCDF) facilities, Singapore. It is a high-fidelity disaster area meant to simulate collapsed buildings after a massive earthquake. Figure 3 shows the test area, which is approximately 8 m × 24 m (192 m^2^) organized as a grid of cells of 2 m × 2 m. This area is composed of two parts, a simulated two floors building partially collapsed (rows 6–13), and a simulated total collapse (rows 1–5). In rows 6–13 there are some accessible and stable paths for rescuing operations, while rows 1–5 represent a totally collapsed area with no accessible rescue paths. 

#### 2.4.2. *Experimental Protocol*

No environmental and structural information about the simulated disaster area was available before starting the experiment. At least 30 min before starting the sensor-based rescue experiment, a person entered the area and randomly hid inside the rubble, simulating an unconscious earthquake casualty. The casualty position had to be estimated within a 2-h time limit, without directly accessing the rubble. However, tools could be inserted through the gaps to acquire data under the rubble. After scanning the entire areas, the position of the casualty had to be estimated. The acceptable identification area consisted of a square of 4 m × 4 m, a 4 cell square. The entire experimental session lasted three days and consisted of three trials per day (a morning, an afternoon, and an evening trial), or nine trials in total. 

## 3. Results and Discussion

### 3.1. Experimental Results

In Table 1, the time needed to detect the casualty in each trial is shown, about one hour on average. We successfully detected the casualty in eight out of nine trials performed. Being fast and precise in casualty detection is a key factor because 80% of survivors are recovered alive if rescued within 48 h. 

**Figure 4:**
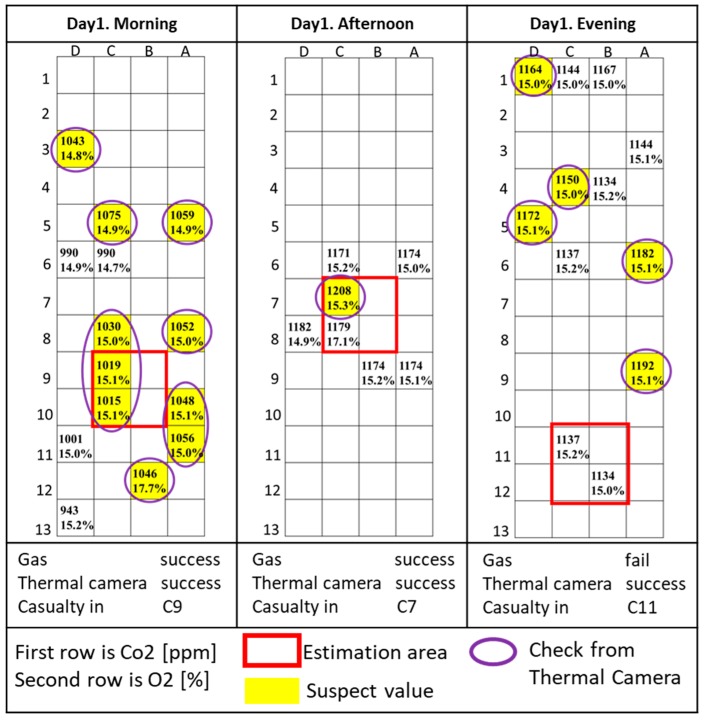
Day 1 results.

**Figure 5:**
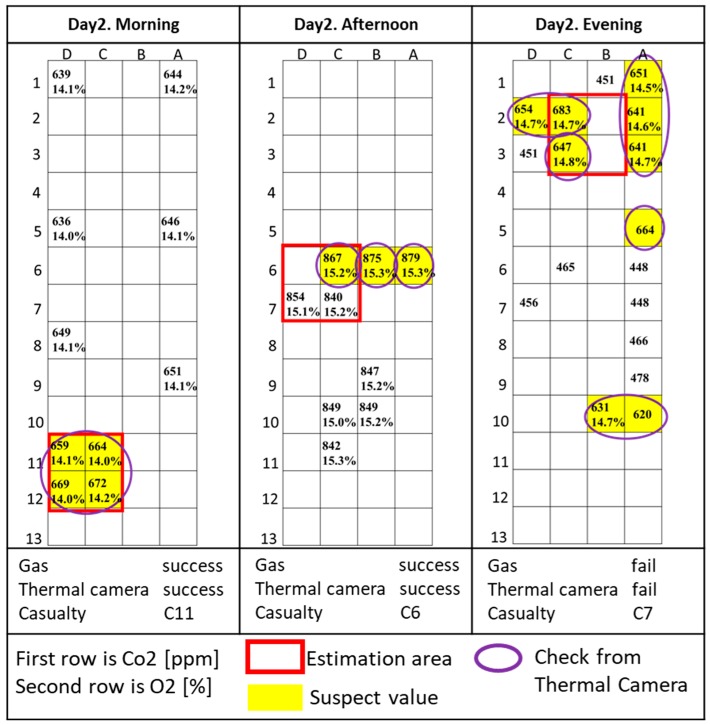
Day 2 results.

**Figure 6:**
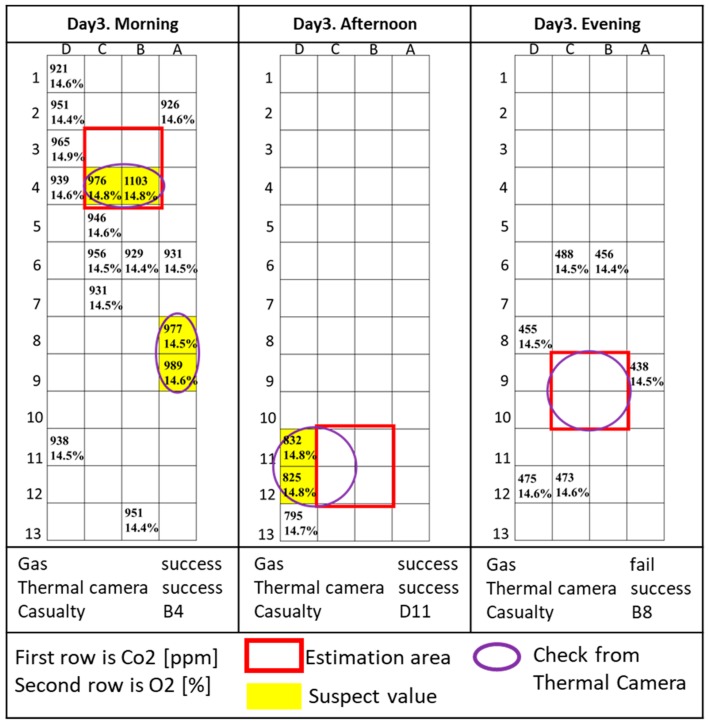
Day 3 results.

The results of each trial are shown Figure 4, Figure 5 and Figure 6 and described and commented on in the rest of this section. O_2_ is measured as concentration, while CO_2_ is in parts-per-million (ppm). Because the CO_2_ sensor is not calibrated, the CO_2_ data do not represent the real concentration and the absolute measured values in each trial vary widely depending on the time the measurement was taken and the environment around the site. For this reason, relative variations of CO_2_ during trials were considered, and further confirmation from a rescuer or other sensors was required to verify the presence of the casualty in that specific area. The areas with relatively high levels of CO_2_ are indicated in yellow. Areas manually checked with a thermal camera are circled in purple.

Figure 4 shows the results of the first day’s trials.

**Day 1, morning trial:** The gas sensor located several possible locations for the casualty. The reason for those abnormal concentrations is that the person reached the center of the site through tunnels in the test sites (C5, A5, A8, A10, and A11 are sections of the same tunnel). The thermal camera images confirmed the presence of the casualty in the estimated area indicated by the red square, in the square composed by cells B9, C9, B10, and C10. 

**Day 1, afternoon trial:** The gas sensor identified an area with a peak CO_2_ concentration and the thermal camera confirmed the presence of the casualty in the area indicated by the gas sensor data, in the square composed by cells B7, C7, B8, and C8.

**Day 1, evening trial:** In this test, the casualty was located in the square composed by cells B11, C11, B12, and C12, using only the thermal camera. The gas sensor did not work properly because the affected area is a large area in which the wind could easily change the CO_2_ concentrations, so the presence of a casualty did not significantly change the CO_2_ concentration in this situation. This test was useful to analyze the factors that can lead to localization failures when using a gas sensor. However, this kind of area can be easily searched by a rescue team or a rescue dog because it is near the boundaries of the disaster area, outside the collapsed structure. 

Figure 5 shows the results of the second day trials.

**Day 2, morning:** Both the gas sensor and the thermal camera located the casualty. The C11, D12, C12, D12 area is part of a corner in which the gas concentration was unusually high, and the camera could be inserted through a hole in the rubble to verify the presence of the casualty.

**Day 2, afternoon:** Both the gas sensor and the thermal camera located the casualty in the square composed by cells C6, D6, C7, and D7 that was beside a wall in a corridor where the gas sensors and the camera could be placed. It is important to note that, in this case, the gas sensor detected a high concentration of CO_2_ in the whole corridor, so the exact position of the casualty could only be confirmed with a thermal camera.

**Day 2, evening:** This was the only trial in which the sensor system failed to locate the casualty. A high concentration of CO_2_ was found in the area around B2, C2, B3, and C3, but the presence of many obstacles obstructing the view made verification via thermal camera impossible. This area is a maze of corridors in a semi-closed area with low air circulation, with the possible presence of grass and animals that might raise the concentration of CO_2_. Moreover, the corridor in C2 was not reachable by gas sensors on the telescopic pole. 

Figure 6 shows the results of the last day’s trials.

**Day 3, morning:** The gas sensor found a high CO_2_ concentration very close to the casualty. However, the presence of many obstacles obstructing the view made verification via thermal camera impossible, so the casualty was located in the square composed by cells B3, C3, B4, and C4 based only on the gas sensor data. 

**Day 3, afternoon:** The casualty was located very fast because the gas sensor measured a relatively high level of CO_2_ in the square composed by cells B11, C11, B12, and C12 and the thermal camera confirmed the presence of the casualty through a hole in the corridor. 

**Day 3, evening:** The casualty was located in the square composed by cells B9, C9, B10, and C10 using only the thermal camera. The data from the gas sensor were corrupted because of hardware problems on the gas sensor board. 

### 3.2. Evaluation of the Gas Sensor and Thermal Camera

**Figure 7:**
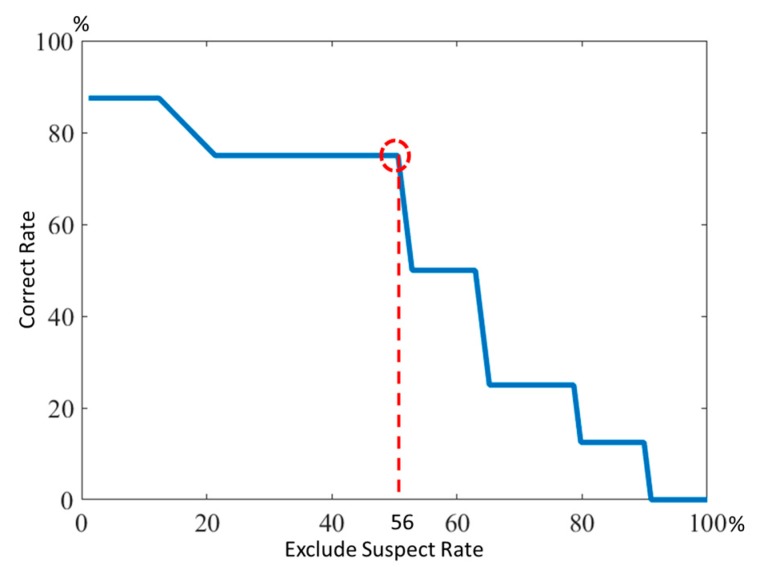
Correct rate and exclude suspect rate.

O_2_ measurements were not useful to determine the presence of life under the rubble. CO_2_ measurements were highly correlated with the possible position of the casualties; however, the CO_2_ sensor failed to locate the casualty in three trials out of nine, one time due to hardware problems and the other times due to environmental conditions. The thermal camera failed to locate the casualty in two trials out of nine, confirming that, although visual analysis is useful, a multi-sensor system is more robust due to sensor redundancy and complementarity. Figure 7 shows the relationship between high casualty localization rate and high casualty presence exclusion rate depending on the CO_2_ threshold. Areas with a high casualty localization rate indicate that the possible presence of the casualty is high, while a high casualty presence exclusion rate indicates areas in which the possibility of presence of the casualty can be reasonably excluded, and so do not need to be cross-checked with the thermal camera. From these empirical data, a method can be devised to estimate a reasonable CO_2_ absolute threshold, correlated with a high casualty localization rate but also with a high casualty presence exclusion rate.

The closest point to (100%, 100%) was found with a CO_2_ threshold of 27 ppm, leading to a reduction of the possible casualty presence area to 44% of the total area and significantly shortening the search and rescue operations. The sensitivity of the CO_2_ sensor is 75% and specificity is 53.1%, as shown in Table 2. 

### 3.3. Evaluation of Microphone and Audio Processing Algorithm

**Figure 8:**
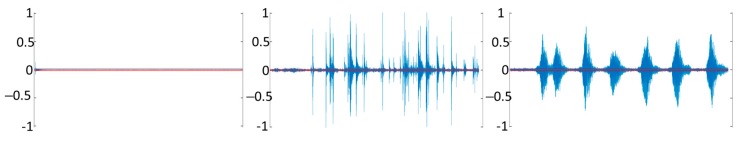
GUI of sound recognition.

In all the experimental trials, the casualty was supposedly unconscious. Therefore, the person did not speak or produce other sounds such as scratching during the whole trial. However, in real disaster scenarios, there are cases in which the casualty is not unconscious and can produce sounds. For this reason, an algorithm for the detection of sounds that might be related to the presence of a casualty was designed and tested. The hardest problem was to make the algorithm less sensitive to background noise. A disaster site is often a noisy environment, with people searching for victims, vehicles, and various natural and artificial sounds. A dynamic threshold for the classification between a possible sign of life and background noise, based on the average level of sound in the area, was proposed. Of course, this method implies that in extremely noisy environments the detection of feeble sounds will not be possible. However, in this way the system is more robust and automatically rejects sounds that are not linked with the presence of casualties, reducing the number of sounds that must be listened for to check the presence of casualty in a specific area. In particular, speech has characteristic features that were used to separate it from other suspect noises. Figure 8 shows the results of the Day 3 afternoon test, in which we spoke directly to the casualty after locating them to test the audio recognition system. The microphone was placed on a telescopic pole and inserted in a hole in the same corridor where the person was detected by using the gas sensor and the camera. Then, we asked the casualty to perform three different tests: to not move and stay in silence while we talked outside, to call for help at a low volume inaudible by the human ear from outside, and to simply scratch on the ground. Audio detection results are shown in Figure 8. Moreover, we detected an unwanted cough, confirming the presence of the casualty, in the area with a high level of CO_2_ during the Day 1 afternoon trial, and another suspect noise during the Day 2 afternoon trial, when the person moved into the corridor. 

The result of audio detection performance evaluation is shown in Table 3. The proposed algorithm can automatically differentiate the sound data and save it in different folders. In Table 3, the first row α/β represents the correct sound identification rate, where β is the total number of automatically classified sound files present in each category folder and α is the correctly classified number of sound files, which were validated manually.

The correct voice recognition rate is 89.36% in a noisy environment. The correct classification rate for human-related suspect noise, including scratching and coughing, is 93.85%. Therefore, using a microphone in connection with other sensors would be beneficial for the detection of casualties. 

## 4. Conclusions

In this study, a new sensor system for detecting human presence under rubble was proposed and tested. The effectiveness of each sensor was evaluated and confirmed. A CO_2_ sensor can provide useful information to locate a casualty, but an O_2_ sensor does not. A voice recognition algorithm based on SVM was also tested and from the results obtained it was confirmed that using the microphone would be of great benefit in the detection of casualties. This system has some limitations; for example, the gas sensor is difficult to use in open spaces due to stronger airflow affecting the CO_2_ concentration. A sensor system using only a thermal camera is not robust because some areas cannot be directly accessed using a telescopic pole or directly observed due to the presence of obstacles. 

In future work, a sensor system should be developed that includes multiple sensors, such as microphones and gas sensors, to be distributed in the area by the rescue team to alert them if one measures signs of a casualty under the rubble. 

This kind of distributed sensor system can also be used in search and rescue operations with robotic aids that can release such sensors in areas inaccessible to or very risky for human rescue teams.

## Figures and Tables

**Table 1:** Global results of the tests.

TEST	Execution Time	Result
Day 1 morning	1 h 35 min	Success
Day 1 afternoon	56 min	Success
Day 1 evening	1 h 25 min	Success
Day 2 morning	33 min	Success
Day 2 afternoon	50 min	Success
Day 2 evening	1 h 12 min	Failed
Day 3 morning	2 h 13 min	Success
Day 3 afternoon	20 min	Success
Day 3 evening	31 min	Success

**Table 2:** Evaluation of gas sensor system.

	Predicted Condition Positive	Predicted Condition Negative
Condition positive	6	2
Condition negative	38	43

**Table 3:** Evaluation of microphone.

TEST	Human Voice	Suspect Noise	Noise
Test Day 1 afternoon	87.5%	89.36%	100%
Test Day 2 afternoon	89.4%	91.21%	100%
Test Day 3 afternoon	90.6%	98.18%	100%
Average	89.36%	93.95%	100%
